# Examining a Psychological Intervention Dataset on Entrepreneurial Attitudes of Undergraduate Accounting Students

**DOI:** 10.3389/fpsyg.2022.948978

**Published:** 2022-07-29

**Authors:** Robinson Onuora Ugwoke, Obioma Vivian Ugwoke, Edith Ogomegbunam Onyeanu, Tijani Ahmed Ajayi

**Affiliations:** Department of Accountancy, Faculty of Business Administration, University of Nigeria, Enugu, Nigeria

**Keywords:** dataset, entrepreneurial attitudes, online REBC, REBT, undergraduate accounting students, Telegram application

## Research Summary

As entrepreneurial intentions and ownership of businesses among students continue to rise, it is essential for students to develop the right entrepreneurial attitudes in order to run their businesses successfully. Accounting students' entrepreneurial attitudes are said to be positively related to their desire to become entrepreneurs. This study aimed at examining the dataset from our study which focused on the effect of online rational emotive behavior coaching (online REBC) delivered *via* Telegram application on entrepreneurial attitudes among Nigerian undergraduate accounting students. We studied a sample of 133 undergraduate accounting students divided into two research groups (online REBC group, *n* = 67; waiting-list control group, *n* = 66). Data collection was accomplished by using an entrepreneurial attitudes scale developed for students. Posttest data from the online REBC group showed significant improvement in entrepreneurial attitudes (i.e., achievement, innovation, personal control, and self-esteem) of students compared to a waiting-list control group. Follow-up data also showed sustained effects of the online REBC group on entrepreneurial attitudes of the students when compared to a waiting-list control group. Participants reported being satisfied with this online REBC intervention.

## Introduction

There has been evidence that social media and secured messaging applications can be used to deliver online interventions to a large group of people (e.g. Obiweluozo et al., 2021; Okeke et al., 2021). It has been demonstrated that online intervention is as effective as other treatment formats in supporting students (Donker et al., 2015; Dear et al., 2019). Furthermore, research has demonstrated that telegram-delivered interventions are as effective as in-person interventions and could improve participants' quality of life (Faraji et al., 2020). Considering that a telegram is an effective tool for the delivery of online courses (Iqbal et al., 2020), it is considered a suitable tool for use in the present study. The telegram is one of the cloud-based messaging and social media applications that enables its users to communicate with one another through mobile data or WiFi connections (Kamat, 2021). Individuals are able to communicate through end-to-end encrypted messages and to distribute electronic contents and media *via* the Telegram application. Telegram can be used to create groups containing about 200,000 people, as well as for video calling, voice calling, sharing photos, audio files, and videos (Kamat, 2021). Therefore, the objective of the current research was to investigate the effect of online rational emotive behavior coaching (online REBC) delivered *via* Telegram on entrepreneurial attitudes among undergraduate accounting students. The current study also aimed to investigate whether the effect of the online REBC delivered *via* Telegram on entrepreneurial attitudes among undergraduate accounting students would be sustained at follow-up. Lastly, this study aimed to investigate participants' satisfaction with the online REBC they received.

### Students' Entrepreneurial Attitudes, Coaching, and Satisfaction With Coaching

As entrepreneurial intentions and ownership of businesses among students continue to rise, it is essential for students to develop the right entrepreneurial attitudes in order to run their businesses successfully (Daze, 2021). There are two main approaches taken when it comes to the nature of attitudes. In one view, attitude is seen as a unidimensional construct and can be sufficiently understood and represented only by affective response (Fishbein and Ajzen, 1975). The second view, referred to as the tripartite model, states that all things can be perceived in terms of three types of responses: affect, cognition, and conation (Chaiken and Stangor, 1987; Shaver, 1987; Robinson et al., 1991). We also agree that attitude is a synthesis of affect, cognition, and conation components. The cognitive component of attitude is composed of the thoughts and beliefs that one holds concerning an attitude object. The affective component of attitude is composed of the way one feels about the object, either positive or negative. The conative or behavioral component of attitude is concerned with a person's intentions and predispositions in regards to how they wish to behave toward the object (Robinson et al., 1991). According to Reyad et al. (2019), entrepreneurial attitudes include cognition of skills and intentions to start a business. Borasi and Finnigan (2010) stated that these entrepreneurial attitudes are vision, taking advantage of opportunities, handling risk, working with resources, and dealing with growth. Reed (2021) refers to these attitudes as passion, courage, flexibility, and a strong work ethic and integrity.

Robinson et al. (1991) described entrepreneurial attitudes as a construct consisting of a number of characteristics, including achievement in business, innovation in business, perceived personal control of business outcomes, and perceived self-esteem in business, which are each composed of three components, namely, affect, cognition, and conation. Following this, these authors (i.e., Robinson et al., 1991) developed the Entrepreneurial Attitudes and Orientation (EAO) scale, which comprises of 75 Likert-type items and four subscales (achievement, innovation, personal control, and self-esteem). In their view, the term achievement refers to tangible accomplishments that can be attributed to the starting and expansion of a business venture by an individual. Innovation in business relates to a person's ability to interpret and respond to business activities in unique and novel manner. Perceived personal control relates to a person's perception of his or her level of control and influence over the direction of a business. Perceived self-esteem refers to the level of self-confidence and perceived level of competence that an individual has in regard to their business endeavors (Robinson et al., 1991). The EAO questionnaire was developed as a response to concerns that other approaches provided weak arguments for distinguishing or defining entrepreneurial characteristics. Prior research has demonstrated the adaptability and validity of the EAO scale to varying populations (e.g., Robinson et al., 1991; Tan et al., 1996; Fernandez et al., 2015), and it is considered a suitable tool for assessing the entrepreneurial attitudes of students. Therefore, we adopted the conceptualization of entrepreneurial attitudes by Robinson et al. (1991) in this present study.

Students' entrepreneurial attitudes can contribute to their psychological wellbeing (Grichnik et al., 2010; Musiiwa et al., 2019). In the context of rational emotive behavior therapy approach, we can infer that tolerance for risk is essential for fostering a positive entrepreneurial attitude. It follows, therefore, that irrational thoughts can lead to a lack of risk tolerance (Ellis, 1962, 1994) and this can ultimately lead to a negative entrepreneurial attitude. Thus, it can be argued that positive emotions stimulate cognition, intuitiveness, and analytical thinking contribute to the emergence of entrepreneurial attitudes (Su et al., 2020). Rational positive emotions can help individuals deal with uncertainties when establishing their businesses (Su et al., 2020). On the other hand, the assessment and exploitation of entrepreneurial opportunities are negatively impacted by negative emotions (Grichnik et al., 2010). Chen et al. (2021) describes the mechanism underlying irrational positive emotions and the development of rational entrepreneurial cognition by stating that positive emotions can assist individuals in improving their entrepreneurial cognition, which, in turn, allows them to increase their entrepreneurial intentions. As indicated by Musiiwa et al. (2019), activated unpleasant emotions have significant negative impacts on students' entrepreneurial attitudes, which in turn affect their entrepreneurial intentions. Therefore, students who have negative entrepreneurial attitudes may be less likely to succeed as entrepreneurs (Musiiwa et al., 2019). It is thus possible to extend rational emotive behavior coaching (REBC; Kodish, 2002), based on the theory of rational emotive behavior therapy (REBT; Ellis, 1962, 1994), to coach students on how to develop the right entrepreneurial attitudes to run a successful business. The REBT approach provides a way to identify and oppose irrational beliefs arising from illogical and unhelpful thinking (Ellis, 1994). Neenan and Dryden (2002) introduced the concept of coaching as a development-focused relationship with a specially trained coach who provides direction to a client on their goals and assists them to achieve their full potential. According to Prater (2021), coaching challenges individuals to develop their strengths, to identify and mitigate their weaknesses, and to grow as individuals. The use of coaching can help individuals increase their emotional wellbeing and accomplishments (Palmer and Gyllensten, 2008; Croswell, 2020). The benefits of rational emotive behavior model of coaching include observation of students' progress by coaches, constructive feedback and monitoring of additional progress by students (Criddle, 2007).

Participants' satisfaction with coaching, especially rational emotive behavior therapy-based coaching (REBT-based coaching) is an understudied aspect of the program. However, a recent study by Wiginton and Cartwright (2020) demonstrated that participants were very satisfied with their coaching encounter and equally agreed that it contributed to their personal growth and success. Also, a more recent study by Okeke et al. (2021) reported that participants responded very positively to their REBT-based coaching intervention and the participants indicated that the coaching intervention they received was satisfactory. Another recent study suggests that participants of REBT-based coaching are satisfied with its effectiveness and delivery approaches (Onyishi et al., 2021). Therefore, the objective of this study was to examine the effects of online rational emotive behavior coaching (online REBC) on the entrepreneurial attitudes of undergraduate accounting students. We also aimed to examine whether participants were satisfied with the online rational emotive behavior coaching that focused on improving their entrepreneurial attitudes.

### Rationale for Investigating Accounting Students

After graduating from University in many parts of Sub-Saharan Africa, graduates find it difficult to get jobs (Musinguzi et al., 2022). Due to the large number of graduates and the limited amount of existing job openings, the conventional image of University graduates seeking public or private sector employments is becoming unimpressive in today's highly competitive environment (Reyad et al., 2018). Yet, it has been a common practice for Nigerian universities to nurture graduates who are better suited for white-collar employments rather than those with entrepreneurial interests and skills (Osakede et al., 2017; Uche, 2017). In Nigeria, most universities have not made significant efforts to change this unfortunate condition and most lecturers consider students who combine entrepreneurial activities with their studies as unserious students (Uche, 2017). The universities can address unemployment and skilling issues that inhibit students from indulging in entrepreneurship, but most of them have continued to encounter several obstacles in enhancing student entrepreneurship including a lack of funding and limited capacity in a rapidly evolving entrepreneurial ecosystem (Musinguzi et al., 2022). To be successful entrepreneurs, students need hands-on practical experiences and problem-solving-centered learning instead of just lecture-based course tutorials (Osakede et al., 2017; Musinguzi et al., 2022). Thus, Uche (2017) argues that the goal of Nigerian University students should go beyond graduating and being workforce ready; the emphasis should be on becoming graduates who are job creators.

According to Dragan et al. (2018), there is a potential link between accounting students' entrepreneurial skills and attitudes and the University curriculum. Melin and Abdullah (2017) found that accounting students' entrepreneurial attitudes are positively related to their desire to become entrepreneurs. The authors also discovered that accounting students' perceived social pressure or norms negatively affect their interest in pursuing entrepreneurship as a career path (Melin and Abdullah, 2017). Asonitou and Kavoura (2019) argued that it is crucial to recognize the role of emotions in developing accounting graduates' skills and the formation of experienced entrepreneurs. Apart from family factors, Shamsuddin et al. (2018) discovered that education and personal attributes can have an impact on accounting students' intention to pursue entrepreneurship as a career path. Additionally, with the appropriate support, accounting students can demonstrate a strong desire to pursue entrepreneurial careers (Shamsuddin et al., 2018). Accounting students' competencies in both personal and professional life can be honed through an entrepreneurship-based learning experience, resulting in increased accounting knowledge, professional growth, and, most importantly, the development of positive entrepreneurial attitudes (Moreira et al., 2020).

According to Reyad et al. (2018), it is critical to foster entrepreneurial cognitive skills (such as communication abilities, logical thinking abilities, problem-solving abilities, and creative thinking) among University accounting students in order to increase their capacity for entrepreneurship start-up and long-standing business ownership. According to Reyad et al. (2019), the acquisition of entrepreneurial skills (critical thinking, innovation, risk taking, autonomy problem solving, and need for achievement) can influence accounting students' entrepreneurship attitudes. After discovering that social media has a significant influence on accounting students' entrepreneurial intentions, Ahmed et al. (2019) proposed the use of social media tools to shape and support accounting students' entrepreneurial intentions and attitudes. The current research focus on promoting positive entrepreneurial attitudes among accounting students is crucial because according to Lam et al. (2017), positive entrepreneurial attitudes play a significant role in influencing the career aspirations of accounting students. Accounting students with a more positive attitude toward entrepreneurship were more likely to aspire to collaborate in an existing public accounting firm or to be the co-founder of their own public accounting firm, according to finding by Lam et al. (2017).

The extent to which these students can achieve entrepreneurial success is shown to be affected by their entrepreneurial attitudes (Harris and Gibson, 2008; Byabashaija and Katono, 2011; Soomro et al., 2020; Majeed et al., 2021) and thus, warrants exposing them to the REBT-based coaching approach which has been demonstrated to be effective in various research contexts. For instance, it is possible to change negative values through REBT-based coaching, according to finding by Abiogu et al. (2021). According to research by David and Cobeanu (2016), REBT-based coaching is associated with a reduction in participants' mood and an improvement in their performance. A study by David et al. (2016) revealed how coaching could positively affect the performance of business managers. The use of REBT-based coaching increased the self-efficacy of financial advisors, according to study by Pousa and Mathieu (2015). Additionally, an investigation into the effectiveness of a REBT-based coaching program was conducted on a group of middle managers in the telecommunications industry. The coaching program improved the coaching skills and rational attitude of the managers and reduced their irrational beliefs (David and Matu, 2013). Ezenwaji et al. (2019) found that when the undergraduate students received REBT-based coaching, their levels of burnout significantly decreased. REBT-based coaching facilitates the clients' ability to manage stress at work, according to research finding by Ugwuanyi et al. (2021). But, it has not been studied whether online REBT-based coaching (online REBC in particular) affects entrepreneurial attitudes of University students. Therefore, this research examines the dataset on the effects of an online REBC intervention on entrepreneurial attitudes (achievement, innovation, personal control, and self-esteem) among undergraduate accounting students.

## Methods

### Study Participants

Study participants were 133 undergraduate accounting students selected randomly from federal and state universities located in the south-east region of Nigeria. In the preliminary assessment stage using purposive sampling, we identified 368 undergraduate accounting students by targeting students' social media pages at various universities. Upon identifying potential participants and assigning them into strata based on their baseline characteristics, the assignment of each of the participants to either the online REBC group or waiting-list control group was performed using simple randomization procedure as suggested by Faul et al. (2007) and Nair (2019). Participants' willingness to participate in the study was taken into account before implementing the simple randomization procedure. Based on the Gpower sample calculator program (Faul et al., 2007), it was shown that at least 56 students are required for this research to observe a statistical power of 85% at 0.05 level of probability and 0.25 pre-determined effect size, critical *f* of 3.08, and correlation of 0.09. Furthermore, a range of 10–15 participants in each study group in a group coaching program is deemed sufficient by Beene (2020) and Sutton (2022). As this study involved two groups, we continued to recruit participants until we had recruited the first 133 eligible participants. As we had also funded the study ourselves, we were unable to surpass this number of participants in order to make best use of the resources available to us. In all, 67 participants were randomized to the online REBC group, while 66 were randomized to a waiting-list control group based on random numbers generated by the Random Allocation Software (RAS) program (Saghaei, 2004). This automated randomization approach enabled the researchers to ensure that there was no knowledge at the time of the recruitment as to which group a participant would be assigned (an approach known as concealment) in order to minimize selection bias in the study as suggested by Hariton and Locascio (2018). All participants were informed that they could discontinue from participating in the research study at any time, without penalty or loss of benefits. Participants were encouraged to notify the research team if they desire to withdraw from the study. As part of the withdrawal process, we told them that participants withdrawing from the study may be asked to inform the research team of the reason(s) for leaving in order to help the research team to recognize potential problems related to the conduct of the study. The participants were made aware of the possibility that the researchers might terminate an individual's participation in the research study for reasons such as to guard them against undue risks or risks with a demonstrated lack of benefits, as well as uphold the integrity of the data being collected.

The reason for the use of a waiting list control group is that psychologists consider it to be a cost-effective and ethical alternative group compared to a no-treatment control group when studying the effects of psychological interventions (VandenBos, 2007; Hesser et al., 2011; Kinser and Robins, 2013; Schimelpfening, 2021). Also, using a waiting-list control group is necessary since providing a facade intervention might be unethical, and it is held that practitioners may not deliberately assist clients with a treatment that they recognize will not work (Grohol, 2014). A waiting-list control group has been considered by Gallin and Ognibene (2012) as a group consisting of participants who are aware of the fact that they are not receiving experimental treatment but will get the opportunity to receive such experimental treatment at a future date. A waiting-list control group can serve two aims, according to Schimelpfening (2021). First, it provides a comparison group for determining if a treatment intervention is effective for the active experimental group. Second, the reason for having a waiting-list control group is that it provides researchers with the opportunity to isolate an independent variable and at the same time study its effect on dependent factors from an unbiased point of view (Schimelpfening, 2021). To ensure that the participants on the waiting-list were not left untreated and/or to tackle the ethical issues of withholding treatments from research participants, we ensured that they were contacted, they consented, were randomized, measured, and made aware of the fact that they will eventually receive the same experimental treatment at later date after the experimental group has already received it.

### Material

The questionnaires below were evaluated by three experts for the appropriateness of the items.

#### Entrepreneurial Attitudes Scale (EAS)

At every stage of data collection (Time 1, Time 2, and Time 3), students received electronic copies of the questionnaires to complete and return to the researchers. The entrepreneurial attitudes of the students were assessed using a Likert-type scale of 75 items adapted from Robinson et al. (1991) to our research context. The instrument has four subscales, namely achievement (23 items), innovation (26 items), personal control (12 items), and self-esteem (14 items). The items were scored on a 10-point scale in which scores from 1 to 5 implied strongly disagree to slightly disagree whereas scores from 6 to 10 implied slightly agree to strongly agree. Sample item include: For business opportunities, I am willing to sacrifice my personal comfort. Those with higher scores had more positive attitudes toward entrepreneurship. Note that some items were reverse scored just as in Robinson et al. (1991). For their original scale, Robinson et al. (1991) reported Cronbach's alphas of 0.70, 0.73, 0.84, and 0.90 for personal control, self-esteem, achievement, and innovation, respectively. Cronbach's alpha for the achievement, innovation, personal control, and self-esteem subscales were 0.82, 0.94, 0.83, and 0.95, respectively in the present study.

#### Socio-Demographic Profile

Socio-demographic information was collected using this instrument. Information collected includes age, gender, ethnicity, and years of study.

#### Coaching Satisfaction Scale (CSS)

To assess participant satisfaction with the REBC (Time 2 and Time 3), an adaptation of Ginsburg and Drake's (2002) instrument was made. It was called *Coaching Satisfaction Scale* (CSS). Eight items were adapted to our research context. Example of item in the CSS is: Having experienced the benefits of this coaching intervention, I would recommend it to others. Students were asked to rate each item statement from 1 (strongly disagree) to 5 (strongly agree). Cronbach's alpha for the CSS was 0.82 in the current study.

#### Additional Reliability for the Measures

Pearson's r was used to find evidence of test-retest reliability for both the EAS and CSS (Heidel, 2022a). Researchers can demonstrate the test-retest reliability when the *p*-value is <0.05 and the Pearson correlation coefficient is >0.7, according to Heidel (2022a). The EAS scores showed strong correlation over time for the achievement (Pearson's *r* = 0.99, *p* = 0.000), innovation (Pearson's *r* = 0.98, *p* = 0.000), personal control (Pearson's *r* = 0.98, *p* = 0.000), and self-esteem (Pearson's *r* = 0.99, *p* = 0.000) subscales. The CSS scores also showed strong correlations over time (Pearson's *r* = 0.98, *p* = 0.000). We determined the inter-scorer reliability (Shrout and Fleiss, 1979; McLeod, 2007; Landers, 2015) for the EAS and CSS using One-Way Random Effects Intraclass Correlation (ICC). According to Koo and Li (2016), ICC values below 0.5 indicate poor reliability, those between 0.5 and 0.75 indicate moderate reliability, those between 0.75 and 0.9 indicate good reliability, and those over 0.90 indicate excellent reliability. In the EAS, we found ICC values of 0.98, 0.96, 0.91, and 0.99 for the achievement, innovation, personal control, and self-esteem subscales respectively, whereas in the CSS we found an ICC value of 0.98, indicating excellent inter-scorer reliability. According to McLeod (2007), test-retest reliability and inter-scorer reliability allow researchers to establish external validity.

In order to establish internal reliability, the split-half method was employed to ensure that all parts of the instruments contributed equally to the attributes that were being measured (McLeod, 2007; Heidel, 2022b). Heidel (2022b) proposed that a Guttman Spef-Half coefficient of 0.80 or greater can be considered as an acceptable level of reliability. For the EAS, the Guttman Splif-Half coefficients were 0.99, 0.89, 0.84, and 0.88 for the achievement, innovation, personal control, and self-esteem subscales respectively, and for the CSS, it was 0.98.

### Study Procedure

The study was conducted using a randomized controlled trial (RCT) design. The RCT design is an experimental design that gives researchers the ability to randomly assign research subjects to one of two groups wherein one group (the experimental group) receives the intervention to be tested, while the other group (the comparison or control group) receives an alternative treatment (Kendall, 2003). It is the analysis of results obtained from the two groups in the trial that allows for the evaluation of the effectiveness of the intervention (Kendall, 2003; Stanley, 2007). The RCT design is the most rigorous method for determining whether a cause-and-effect relationship exists between the intervention and the outcomes of interest (Kendall, 2003; Stanley, 2007; Hariton and Locascio, 2018). This design was used by Ezenwaji et al. (2019); Abiogu et al. (2021); Okeke et al. (2021), and Onyishi et al. (2021) in their REBT-based coaching interventions, in which participants were randomly assigned to either a treatment group or control group; therefore, we deemed it appropriate to adopt this research design.

Coaches received briefings prior to the kickoff of the program to become familiar with its objectives, evaluate its content and expectations. Participants who volunteered to take part in the study were pretested. In the coaching group, REBC was then delivered through Telegram, whereas students in the control group remained on a waiting-list to begin the intervention 2 weeks after follow-up. They received coaching twice a week between 4:30 and 6:30 pm for 8 weeks, followed by 2 weeks of follow-up (2 sessions per week) 3 months later. The fidelity of the coaching program was evaluated. The treatment fidelity checklist developed by Borrelli et al. (2005) was used for this purpose. An external rater assessed 100% treatment fidelity across all online sessions of the coaching program.

### Summary of Intervention

The online coaching program was based on an adapted version of the rational emotive coaching manual (Otu, 2020). This program is delivered through a Telegram group and focuses on enhancing entrepreneurial attitudes among undergraduate accounting students. Some coaching techniques utilized in the study were goal setting, and alliance building (Kodish, 2002). The coaching intervention also involved the use of a number of rational emotive behavior therapy techniques advocated by Si and Zhang (2017) including cognitive reframing, empirical disputes, functional disputes, and adversity coping statements, which are usable for modifying participants' low tolerance to frustrating entrepreneurship scenarios. According to Zaleskiewicz et al. (2020), mental imagery technique can also be used to improve individual's entrepreneurial attitudes. Motivational and advisory techniques were adapted from Nachreiner et al. (2015) to reinforce participants for their commitments toward improving their entrepreneurial attitudes.

The first and second week sessions involved introducing participants and coaches, familiarizing with coaching, explaining coaching concepts, describing online REBC expectations, and elaborating entrepreneurial attitudes. During these sessions, the researchers alongside the participants used the techniques of motivational interviewing, goal setting and alliance building to familiarize with one another and learn about the essential coaching concepts, online REBC expectations and what entrepreneurial attitudes entails.

During the second and third sessions (3rd and 4th weeks), some coaching techniques were used to improve entrepreneurial attitudes. During these sessions, participants used the mental imagery technique to demonstrate how an individual can deliberately attempt to simulate the future and create images that would be able to create a visual representation of both the positive and the negative outcomes of the entrepreneurial choices they make. The imagery that may be considered positive and pleasant may include those related to developing the business and making money (Zaleskiewicz et al., 2020). As such, visualization of consequences can be understood as a pre-decisional “testing” of different courses of action and what might happen as a result (Ji et al., 2016).

The fifth and sixth lessons (5th and 6th weeks) focused on fostering positive entrepreneurial attitudes. During these sessions, participants learned about coaching techniques (cognitive reframing, empirical disputes, functional disputes, and adversity coping statements—Si and Zhang (2017) and how to apply them in developing and maintaining positive entrepreneurial attitudes were taught to participants. In this case, undergraduate accounting students gained the ability to become entrepreneurs themselves and were motivated to apply the skills they acquired.

In the final two sessions (7th and 8th weeks), we evaluated the improvements in entrepreneurial attitudes achieved through coaching by receiving further feedback from participants and offering reinforcements. During these sessions, motivational and advisory techniques adapted from Nachreiner et al. (2015) were used to reinforce participants for their commitments toward the program. This online REBC program was moderated by four business coaches with doctoral degrees (two men and two women) and more than 5 years of postdoctoral training.

#### Method of Data Analysis

Data were checked for missing variables and assumptions were tested. Because of the statistical dependence of data resulting from group-based interventions, which could amplify the chances of Type I errors when disregarded (Baldwin et al., 2008), repeated measures multivariate analysis of variance was employed for the analysis of the research data on students' entrepreneurial attitudes at the 0.05 level of probability. To avoid any bias in the analysis, demographic variables with significant differences between the two groups may be included as covariates (to run repeated measures MANCOVA) but if all of them are not significant, they will be excluded as recommended by Lund Research (2018) and Ayla (2020). The one-sample t-test and paired sample t-test statistics were used to analyze data concerning online REBC group participants' satisfaction with the coaching program. *Post-hoc* analysis for Group × Time interaction effects was carried out with Sidak in order to see which group experienced improvement on entrepreneurial attitudes just as in recent REBT intervention (Oloidi et al., 2022).

## Results and Discussion

The dataset reveals that there were no significant variations in the socio-demographic profiles of the students: (age in years: χ^2^ = 1.09, *p* > 0.05); (ethnicity: χ^2^ = 0.87, *p* > 0.05); (year of study: χ^2^ = 1.16, *p* > 0.05); and (student gender: χ^2^ = 0.70, *p* > 0.05; see Table 1). In other words, we found none of the demographic factors to be significant, so they had no effect on the outcomes of this study. Hence, in order to analyze the study data on entrepreneurial attitudes, the repeated measures multivariate analysis of variance was used, rather than the repeated measures analysis of covariance.

**Table 1:** Socio-demographic profile of the students.

**1 Demographics of participants**		**Online REBC group *n* (%)**	**Waiting-list control group *n* (%)**	**χ^2^**	***p***
Age	Below 20 years	18 (26.9)	16 (23.9)	1.09	>0.05
	20–25 years	39 (58.2)	42 (62.7)		
	25 years and above	10 (14.9)	8 (11.9)		
Ethnicity	Igbo	34 (50.7)	32 (47.8)	0.87	>0.05
	Hausa	11 (16.4)	12 (17.9)		
	Yoruba	22 (32.8)	12 (17.9)		
Year of study	1st year	18 (26.9)	20 (29.9)	1.16	>0.05
	2nd year	21 (31.3)	18 (26.9)		
	3rd year	17 (25.4)	19 (28.4)		
	4th year	11 (16.4)	10 (14.9)		
Gender	Male	32 (47.8)	29 (43.3)	0.70	>0.05
	Female	35 (52.2)	37 (55.2)		

Table 2 provides the descriptive statistics (means and standard deviations) for the study variables by time and study group.

**Table 2:** Descriptive statistics (means and standard deviations) for the study variables by time and study group.

**Time/EAS subscales**	**Group**	**Mean**	**SD**
Pretest—achievement	Online REBC group	81.91	3.21
	Waiting-list control group	81.03	4.18
Pretest—innovation	Online REBC group	82.10	15.07
	Waiting-list control group	80.77	14.41
Pretest—personal control	Online REBC group	40.81	9.04
	Waiting-list control group	42.55	6.27
Pretest—self-esteem	Online REBC group	42.24	7.96
	Waiting-list control group	40.45	9.74
Posttest—achievement	Online REBC group	188.10	4.32
	Waiting-list control group	80.14	4.09
Posttest—innovation	Online REBC group	213.28	4.82
	Waiting-list control group	79.18	13.72
Posttest—personal control	Online REBC group	99.81	1.31
	Waiting-list control group	41.14	5.49
Posttest—self-esteem	Online REBC group	113.00	0.00
	Waiting-list control group	39.08	9.16
Follow-up—achievement	Online REBC group	188.84	4.42
	Waiting-list control group	78.85	5.29
Follow-up—innovation	Online REBC group	213.82	4.77
	Waiting-list control group	78.59	13.57
Follow-up—personal control	Online REBC group	100.31	1.59
	Waiting-list control group	40.35	5.78
Follow-up—self-esteem	Online REBC group	114.85	3.00
	Waiting-list control group	37.78	9.31
**Time/satisfaction with coaching**			
Posttest—CSS	Online REBC group	35.24	1.09
Follow-up—CSS	Online REBC group	36.51	1.29

*EAS, Entrepreneurial Attitude Scale; CSS, Coaching Satisfaction Scale; SD, Standard Deviation*.

The dataset, based on the test of between-subjects effects, showed that the pre-test scores for the achievement [*F*_(1, 132)_ = 1.85, *p* = 0.176], innovation [*F*_(1, 132)_ = 0.27, *p* = 0.603], personal control [*F*_(1, 132)_ = 1.66, *p* = 0.200], and self-esteem [*F*_(1, 132)_ = 1.34, *p* = 0.249] subscales of the Entrepreneurial Attitudes Scale (EAS) were significantly similar between the online REBC group and control waiting-list control group.

However, posttest data, based on the test of between-subjects effects, revealed significant differences for the achievement [*F*_(1, 132)_ = 21,884.35, *p* = 0.000, Partial η^2^ = 0.99], innovation [*F*_(1, 132)_ = 5,685.94, *p* = 0.000, Partial η^2^ = 0.98], personal control [*F*_(1, 132)_ = 7,248.73, *p* = 0.000, Partial η^2^ = 0.98], and self-esteem [*F*_(1, 132)_ = 4,362.03, *p* = 0.000, Partial η^2^ = 0.97] subscales of the EAS between the online REBC group and the waiting-list control group. The dataset based on the multivariate test suggest that the online REBC intervention significantly improved students' entrepreneurial attitudes compared to a waiting-list control condition [*F*_(11, 121)_ = 5,155.47, *p* = 0.000, Partial η^2^ = 0.99, Wilks' Λ = 0.002]. The Sidak's *post-hoc* for the Group × Time interaction also confirmed these significant differences in students' posttest scores between the two study groups in favor of the online REBC group for the achievement (*Mean difference* = 107.97), innovation (*Mean difference* = 134.10), personal control (*Mean difference* = 58.67), and self-esteem (*Mean difference* = 73.92) subscales of the EAS.

Further, follow-up data based on the test of between-subjects effects also showed that the effects of online REBC program on students' entrepreneurial attitudes—achievement [*F*_(1, 132)_ = 16,911.07, *p* = 0.000, Partial η^2^ = 0.99], innovation [*F*_(1, 132)_ = 5,915.84, *p* = 0.000, Partial η^2^ = 0.98], personal control [*F*_(1, 132)_ = 6,700.39, *p* = 0.000, Partial η^2^ = 0.98], and self-esteem [*F*_(1, 132)_ = 4,149.48, *p* = 0.000, Partial η^2^ = 0.97]—were significantly sustained among participants in the online REBC group compared to the waiting-list control group. The Sidak's *post-hoc* for the Group × Time interaction also confirmed that significant differences exist in students' follow-up scores in favor of those in the Online REBC group for achievement (*Mean difference* = 109.99), innovation (*Mean difference* = 135.23), personal control (*Mean difference* = 59.97), and self-esteem (*Mean difference* = 77.06) subscales of the EAS.

The one-sample *t*-test analysis of the posttest data on satisfaction with coaching indicated that participants in the online REBC group reported significant satisfaction with the online REBC program they received [*t*_(66)_ = 257.50, *p* = 0.000, Cohen's *d* = 1.09, 95% CI = 33.97, 34.50]. According to the paired sample t-test analysis, participants' report of satisfaction with the online REBC program from posttest to follow-up was significantly consistent [*t*_(66)_ = −8.39, *p* = 0.000, Cohen's *d* = 1.24, 95% CI = −1.57, −0.97, *r* = 0.47].

The dataset of this study support those of David et al. (2016), who discovered that coaching programs improved the skills and performance of coachees. Additionally, David and Matu (2013) found that coaching was helpful in improving rational attitudes of participants. This study supports Palmer and Gyllensten's (2008) claim that coaching enhances performance attitudes. It is also in agreement with the study by Ogbuanya et al. (2017), which stated that REBC, a REBT-based coaching intervention, is a time-efficient approach for improving cooachees' ability. Using REBT-based coaching, Agu et al. (2021) found that the quality of efforts demonstrated by coachees increased significantly. The findings of this study also support the findings of Abiogu et al. (2021), who showed that REBT-based coaching reduced negative values significantly in clients. Ntamu (2017) also discovered that coaching improved participants' attitudes, which is consistent with the findings of the present study indicating an improvement in positive entrepreneurial attitudes.

The effect of online rational emotive behavior coaching on entrepreneurial attitudes of undergraduate accounting students was affirmative and participants' were satisfied with the coaching program they received, according to our dataset. The result reported in Okeke et al.'s (2021) REBT-based coaching intervention confirms our data, as participants reported to have been satisfied with the intervention they received. Participants have been reported to be satisfied with REBT-based coaching (Onyishi et al., 2021), which is consistent with the dataset of this study. The dataset of this study is similar to those of Wiginton and Cartwright (2020) who showed that participants were very satisfied with their coaching experiences and equally agreed that the experience contributed to their personal development. Therefore, developing and incorporating the rational emotive behavior coaching program in the undergraduate accounting students' psychosocial support services may go a long way toward building great entrepreneurs. Undergraduate accounting students in Nigerian universities should be offered this program to encourage the development and improvement of positive entrepreneurship attitudes. As the number of University students who launch their own businesses while continuing their studies is increasing every year due to a wide variety of factors, including their desire, generous donations, and need to make a profit (Daze, 2021), it is encouraging that student start-ups receive support from the University. Through experiential learning, students have the opportunity to try new things, put their knowledge into practice, and form valuable relationships. By engaging in hands-on, experiential learning activities, students can develop a number of critical entrepreneurial skills (Daze, 2021).

### Limitations of the Research and Suggestions

Despite its strengths, this study has some flaws. Only quantitative data were used to evaluate the effectiveness of the online REBT-based coaching program. In addition, the research was limited to undergraduate accounting students of federal and state universities, with no involvement of accounting students from private universities. As the control group in this study was waitlisted, it would be informative to compare face-to-face REBC with online REBC in future coaching interventions. Although there are ethical merits to the use of a waiting-list since it enables the same care to be offered to research participants seeking help while allowing non-intervention assessments (VandenBos, 2007; Hesser et al., 2011; Cunningham et al., 2013; Kinser and Robins, 2013; Schimelpfening, 2021), it has been observed that the use of this type of group could result in the overestimation of treatment effects (Grossman et al., 2007; Cunningham et al., 2013). This is because, according to Cunningham et al. (2013), waiting-list control group participants might demonstrate less improvement than participants in an experimental group who are taking therapeutic actions to modify their behaviors. As a result, future researchers may use other types of control groups such as usual care, treatment as usual, and standard of care controls that play a critical role in behavioral intervention studies as recommended by Freedland et al. (2011) to further investigate and substantiate the effects of online REBC on students' entrepreneurial attitudes.

### Practical Implications and Contributions of the Research

Entrepreneurial coaches and their teams can use the results of this study to further understand how students' entrepreneurial attitudes impact their business intentions and business ownership, as well as potential behavioral intervention techniques that can be tailored to assist students in developing positive entrepreneurial attitudes. It is our hope that the insights from this research may also be utilized by school policymakers to develop initiatives to improve students' entrepreneurship skills, as well as to enhance existing entrepreneurship programs for this population. Researchers can use the insights gained from this study to study how entrepreneurial attitudes may influence students' ownership of businesses in developing countries. Many universities need to do more to make sure that all students, in spite of their academic or career prospects, have access to the essential entrepreneurship skills they will need in their future careers. Universities need to remain vigilant in improving the quality of their student body, the content they teach, and their teaching methods (Daze, 2021).

According to this study, online rational emotive behavior coaching appears to benefit undergraduate accounting students by developing their entrepreneurial attitudes. The study contributes to the expansion of the rational-emotive behavior coaching framework and potential application in similar contexts for promoting positive entrepreneurial attitudes among students. The study evaluated the benefits of an online coaching program for undergraduate accounting students from a developing nation, which is a hardly ever studied group. Research into participant satisfaction levels with REBC coaching is limited, but the current study examined this issue, thus adding this knowledge to the literature.

## Conclusion

The dataset suggest that online rational emotive behavior coaching (online REBC) delivered *via* Telegram app can improve entrepreneurial attitudes (i.e., achievement, innovation, personal control, and self-esteem) among undergraduate accounting students. The study opens up opportunity for new knowledge and experiences about potential ways to motivate accounting students to develop positive entrepreneurial attitudes in other Nigerian school contexts. It is expected that future studies will investigate whether online REBC will improve accounting students' money attitudes and trauma. Universities need to do more to make sure that all students, in spite of their academic or career prospects, have access to the essential entrepreneurship skills they will need in their future careers.

## Data Availability Statement

The original contributions presented in the study are included in the article, further inquiries can be directed to the corresponding author.

## Ethics Statement

The studies involving human participants were reviewed and approved by the Research Ethics Committee at the University of Nigeria. This study was conducted in accordance with the ethical standards of the 1964 Helsinki Declaration and its later amendments or comparable ethical standards. Online consent forms were received, read, filled out, and signed by all included participants. The patients/participants provided their written informed consent to participate in this study.

## Author Contributions

RU, OU, EO, and TA conceived the study and equally responsible for the study design and implementation. All authors agree to be accountable for the content of the work.

## Conflict of Interest

The authors declare that the research was conducted in the absence of any commercial or financial relationships that could be construed as a potential conflict of interest.

## Publisher's Note

All claims expressed in this article are solely those of the authors and do not necessarily represent those of their affiliated organizations, or those of the publisher, the editors and the reviewers. Any product that may be evaluated in this article, or claim that may be made by its manufacturer, is not guaranteed or endorsed by the publisher.
